# Successful Use of Intravenous Immunoglobulins in an Obinutuzumab-related Acute Thrombocytopenia

**DOI:** 10.1097/HS9.0000000000000751

**Published:** 2022-07-15

**Authors:** Tobias R. Haage, Alexey Surov, Dimitrios Mougiakakos, Mirjeta Berisha

**Affiliations:** 1 Department of Hematology and Oncology, Otto-von-Guericke University, Medical Center, Magdeburg, Germany; 2 Department of Radiology and Nuclear Medicine, Otto-von-Guericke University, Medical Center, Magdeburg, Germany

Targeted anti-CD20 treatment is effectively used in B-cell neoplasia such as follicular lymphoma (FL). In combination with chemotherapy, the third-generation anti-CD20 monoclonal antibody (mAb) obinutuzumab achieved prolonged progression-free survival as compared to a rituximab-based therapy in patients with FL.^1^ Especially patients with risk factors (age >60 y, >4 nodal sites, elevated lactate dehydrogenase (LDH), hemoglobin <120 g/L, Ann Arbor stage III–IV) leading to an intermediate or high Follicular Lymphoma International Prognostic Index (FLIPI) benefit from obinutuzumab.^2,3^

A 56-year-old female presented with progressive dyspnea and FL, initially diagnosed one week prior to the admission. During an inpatient treatment of a myocardial infarction three weeks preadmission, two drug-eluting stents were implanted in the ramus circumflexus within percutaneous coronary intervention. Hereafter, the patient received an oral dual antiplatelet therapy with acetylsalicylic acid (ASA) 100 mg and clopidogrel 75 mg daily. Incidentally, ascites and an extensive axillary, clavicular, mediastinal, and retroperitoneal lymphadenopathy became apparent (Figure 1). After axillary lymphadenectomy FL grade 1–2 was histologically identified. Ascites was also possibly associated with FL throughout cytological examination. A bone marrow infiltration up to 20% was histologically detected. FLIPI reached 4 points indicating a high risk due to the involvement of >4 nodal sites, a slightly elevated LDH, a hemoglobin <120 g/L, and an Ann Arbor stage IV.

**Figure 1.**
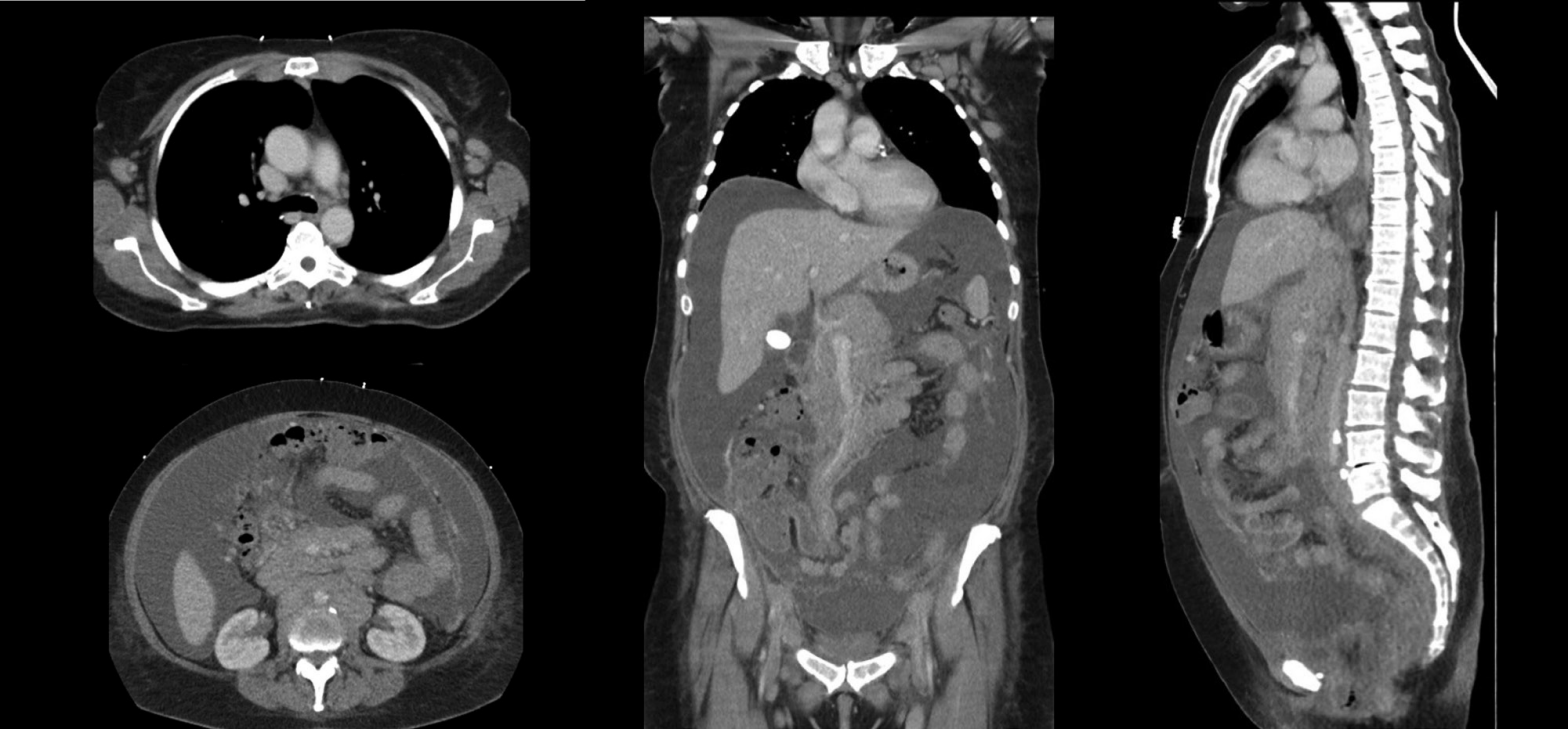
**Computer tomography at initial diagnosis.** Representative sections of computer tomography showing ascites and an extensive axillary, clavicular, mediastinal, and retroperitoneal lymphadenopathy at time of initial diagnosis of follicular lymphoma.

At admission time, the patient complained about progressive dyspnea, edema of the lower legs, an increasing abdominal circumference, and a weight gain of about 6 kg over the last week. Night sweats occurred frequently. Initial blood count analysis was as follows: leukocyte count 11.4 × 10^9^/L (4–10 × 10^9^/L), hemoglobin 109.5 g/L (120–160 g/L), and platelet count 245 × 10^9^/L (150–375 × 10^9^/L). Dyspnea rapidly improved after therapeutic abdominal paracentesis of about 2000 mL ascites and intravenous furosemide therapy. After a pre-phase treatment with prednisolone, we decided to initiate an immunochemotherapy with obinutuzumab and bendamustine. Bendamustine was administered as intravenous infusion at a dose of 155 mg (90 mg/m^2^) on days 1 and 2. Due to the high tumor burden, we decided to split the obinutuzumab infusion to decrease the risk of infusion-related reactions. Thus, obinutuzumab was administered as an intravenous infusion at a dose of 100 mg on day 1 (100 mg absolute, 25 mg/h) and 900 mg on day 2 (900 mg absolute, 50 mg/h, increased in increments of 50 mg/h every 30 min up to a maximum of 400 mg/h).^2,4^ The patient received a premedication with paracetamol 1000 mg orally, prednisolone 100 mg, and clemastine 2 mg intravenously about one hour before obinutuzumab administration on days 1 and 2. Overall, the treatment was well tolerated except for mild chills on day 1 during obinutuzumab application. Within one day after immunochemotherapy, the patient developed a marked thrombocytopenia with a platelet count of 19 × 10^9^/L. Thrombocytopenia was confirmed in the control measurements, platelet aggregates, and fragmentocytes were microscopically excluded. There was no evidence of type II heparin-induced thrombocytopenia (HIT) or disseminated intravascular coagulation (DIC). Type II HIT was less likely because no heparin was administered since myocardial infarction three weeks preadmission. Further, no IgG class antibodies against the complex of platelet factor 4 and heparin were detected. Coagulation parameters remained unaffected over time: prothrombin time (PT) 77% (>70%), international normalized ratio 1.18 (<1.15), activated partial thromboplastin time (aPTT) 27.3 seconds (<34.4 s), thrombin time 14.7 seconds (<18.5 s), and fibrinogen 3.65 g/L (1.5–4.0 g/L). Since no drop of PT or fibrinogen and no prolonged aPTT became apparent, DIC was unlikely. Two days after immunochemotherapy, platelet counts reached its lowest level with 4 × 10^9^/L. The dual antiplatelet therapy was indispensable early after coronary stent implantation, especially to prevent in-stent thrombosis and reinfarction.

Under a close and constant risk-benefit consideration of antiplatelet therapy and bleeding complications, the risk of in-stent thrombosis and reinfarction was considered as leading. In close consultation with and on the recommendation of our cardiological colleagues, we decided to continue clopidogrel and discontinue ASA for 1 day. In addition, three platelet concentrates were in total transfused. The concentration of IgG was slightly reduced with 5 g/L. Suspecting an immune-mediated, obinutuzumab-related thrombocytopenia, intravenous immunoglobulins (IVIg) 10 g daily were administered for 3 days. An additional therapy with corticosteroids was not performed. IgG concentration increased to 6.24 g/L. Platelet counts spontaneously increased on day 4 and completely recovered on day 6 after immunochemotherapy (Figure 2). No signs of bleeding occurred during thrombocytopenia. Missing only one dose of ASA, the patient could be discharged without complications. We refrained from further obinutuzumab administration. The immunochemotherapy was continued on time using rituximab and bendamustine under a close-meshed monitoring of blood parameters. Overall, the patient received two cycles of rituximab and bendamustine with platelet counts remaining unaffected, and therapy is still ongoing.

**Figure 2.**
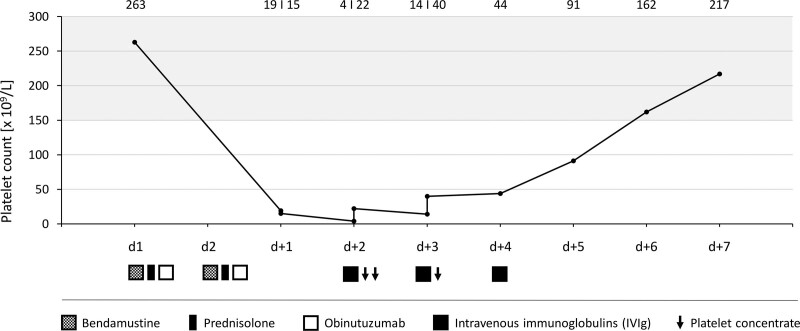
**Time course of platelet count and corresponding treatment.** Platelet counts during immunochemotherapy and post-treatment phase. Bendamustine, prednisolone, obinutuzumab, IVIg, and platelet concentrates were administered as indicated. Platelet counts reached its lowest level two days and completely recovered six days after immunochemotherapy.

In the GALLIUM trial, 11.4% of obinutuzumab- opposed to 7.5% of rituximab-treated patients experienced thrombocytopenia. Among these, 6.1% (obinutuzumab) and 2.7%, respectively (rituximab) developed “severe” thrombocytopenia grade ≥3, as assessed by the National Cancer Institute’s Common Terminology Criteria for Adverse Events.^2^ However, thrombocytopenia is not specifically obinutuzumab-related, but more commonly observed in patients receiving obinutuzumab-based regimens. Thrombocytopenia occurring within 24 hours after immunotherapy was considered as “acute” thrombocytopenia. Thrombocytopenia as well as severe thrombocytopenia most frequently occurred during induction therapy.^2^ Moreover, infusion-related reactions such as an acute thrombocytopenia were most common after the first infusion of anti-CD20 mAb.^2,5^ As recently reported by Sakai et al,^6^ a patient with relapsed FL developed an acute thrombocytopenia during maintenance therapy with obinutuzumab. Platelet counts started to decrease one hour after the end of obinutuzumab administration and dropped at its lowest level four days and partially recovered ten days after administration. Several reports additionally outline the occurrence of acute thrombocytopenia after a rituximab-based treatment in various B-cell neoplasia.^7,8^ However, the pathomechanisms of anti-CD20 mAb-related acute thrombocytopenia are still not completely understood. In a small cohort of patients with chronic lymphocytic leukemia, proinflammatory cytokines such as interleukin (IL)-6 and IL-8 were directly released after the first administration of obinutuzumab.^5^ Thus, cytokine-mediated effects might be plausible. Further explanatory approaches throughout the literature included immune-mediated lysis of CD20 antigen-presenting platelets or complement-activation through circulating soluble CD20 antigen.^9^ Recently, Hinterleitner et al^10^ demonstrated the transfer of programmed death-ligand 1 (PD-L1) from PD-L1 expressing non-small cell lung cancer cells to platelets in vitro and in vivo. Transmission of CD20 to platelets is therefore conceivable, especially as patients with B-cell neoplasia developing anti-CD20 mAb-related thrombocytopenia frequently sharing bone marrow involvement.^5,11,12^ IVIg are used among patients with immune thrombocytopenia to inhibit the immune-mediated degradation of autoantibody-labeled platelets.^13^ Thus, administering IVIg might represent a promising therapeutic approach in anti-CD20 mAb-related (acute) thrombocytopenia, although the specific mechanisms are still unknown.

Obinutuzumab-related acute thrombocytopenia occurring within 24 hours after administration of obinutuzumab is a sudden and severe event. It remains unclear whether complete recovery of platelet counts after obinutuzumab-based therapy in the presented case was solely due to applying IVIg. Nevertheless, IVIg might accelerate platelet count stabilization and recovery in obinutuzumab- or anti-CD20 mAb-related thrombocytopenia in general. However, close-meshed monitoring of platelet counts within patients undergoing an anti-CD20 mAb treatment is recommendable. Particularly since, as in this case, a marked infusion-related reaction does not necessarily precede acute thrombocytopenia.

## ACKNOWLEDGMENTS

We thank the patient for giving her consent. Written informed consent was obtained from the patient for publication purpose.

## AUTHOR CONTRIBUTIONS

All authors provided critical feedback and contributed to shape the article.

## DISCLOSURES

The authors have no conflicts of interest to disclose.
